# Utilization of Transthoracic Echocardiography and Biochemical Markers in Detecting Cardiomyopathy in Fabry Disease

**DOI:** 10.1016/j.cjco.2025.01.017

**Published:** 2025-01-23

**Authors:** Ashwin Roy, Sophie E. Thompson, James Hodson, Kyaw Zaw Win, Amor Mia Alvior, Max J. Cumberland, Antonio Ochoa-Ferraro, David Oxborough, Tarekegn Geberhiwot, Richard P. Steeds

**Affiliations:** a Institute of Cardiovascular Sciences, University of Birmingham, Birmingham, UK; b Department of Cardiology, University Hospital Birmingham NHS Foundation Trust, Birmingham, UK; c Research Development and Innovation, University Hospitals Birmingham NHS Foundation Trust, Birmingham, UK; d The Westmead Institute of Medical Research, Westmead, New South Wales, Australia; e Department of Inherited Metabolic Diseases, University Hospital Birmingham NHS Foundation Trust, Birmingham, UK; f School of Sport and Exercise Sciences, Liverpool John Moores University, Tom Reily Building, Byrom Street, Liverpool, L3 3AF, UK; g Institute of Metabolism and System Research, University of Birmingham, Birmingham, UK

## Abstract

**Background:**

Fabry disease (FD) is an X-linked lysosomal storage disorder caused by α-galactosidase A enzyme deficiency, resulting in multiorgan accumulation of glycosphingolipid. Cardiac accumulation leads to left ventricular hypertrophy, diastolic dysfunction, fibrosis, and sudden cardiac death. Advances in transthoracic echocardiograms (TTEs) have enabled the detection of subclinical atrial and ventricular cardiomyopathy. Until now, studies assessing changes on TTE in FD have been small and cross-sectional. To understand longitudinal changes, our aim was to quantify trends in TTE parameters, linked to relevant physiological and biochemical parameters.

**Methods:**

A single-centre retrospective study was conducted of 75 FD patients who received longitudinal follow-up care (53% female, 57% on enzyme replacement therapy) between 2011 and 2023.

**Results:**

Longitudinal follow-up care demonstrated increasingly impaired left ventricular global longitudinal strain (GLS), tissue Doppler imaging, and right ventricular systolic function. Atrial changes included increasingly impaired left atrial GLS, greater volumes, and reduced left atrial ejection fraction and fractional area change. A sex-specific increase occurred in indexed left ventricular mass in male patients. Biochemical changes included increases in high-sensitivity Troponin-T and N-terminal-pro-B-type natriuretic peptide levels. A sex-specific increase in the urine protein level and the albumin-creatinine ratio in male patients.

**Conclusions:**

TTE and biochemical trends highlight the gradual and insidious nature of FD progression, and stress the importance of considering multiparametric endpoints, including GLS, atrial function, and biomarkers, when assessing outcome in FD.

## Graphical abstract

**Figure:**
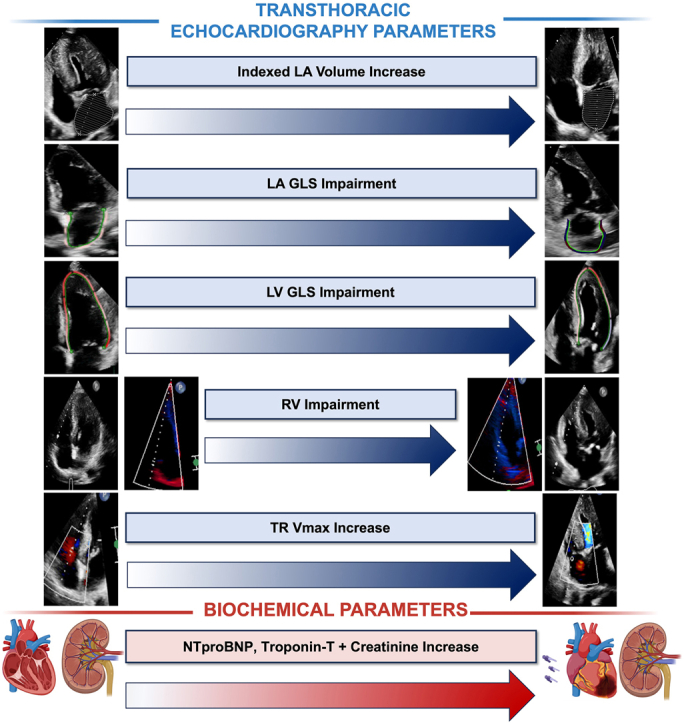

## Résumé

**Contexte:**

La maladie de Fabry (MF) est une maladie de surcharge lysosomale liée à l'X causée par un déficit en enzyme α-galactosidase A, entraînant une accumulation de glycosphingolipides dans plusieurs organes. L'accumulation cardiaque conduit à une hypertrophie du ventricule gauche, une dysfonction diastolique, une fibrose et une mort subite cardiaque. Les progrès des échocardiogrammes transthoraciques (ETT) ont permis de détecter des cardiomyopathies atriales et ventriculaires subcliniques. Jusqu'à présent, les études évaluant les changements sur l'ETT chez les personnes atteintes de la MF étaient de petite taille et transversales. Pour comprendre les changements longitudinaux, notre objectif était de quantifier les tendances des paramètres ETT, liées à des paramètres physiologiques et biochimiques pertinents.

**Méthodes:**

Une étude rétrospective monocentrique a été menée sur 75 patients atteints de la MF ayant bénéficié de soins avec suivi longitudinal (53% de femmes, 57% sous traitement enzymatique de substitution) entre 2011 et 2023.

**Résultats:**

Le suivi longitudinal a montré une altération croissante de la déformation longitudinale globale (DLG) du ventricule gauche, de l'imagerie Doppler tissulaire et de la fonction systolique du ventricule droit. Les changements atriaux comprenaient une altération croissante de la DLG de l'oreillette gauche, des volumes plus importants, et une réduction de la fraction d'éjection auriculaire gauche et de la fraction de raccourcissement de surface. La masse ventriculaire gauche indexée a connu une élévation spécifique au sexe chez les patients masculins. Les changements d'ordre biochimique comprenaient des augmentations des taux de troponine-T à haute sensibilité et du propeptide natriurétique de type B N-Terminal. Une augmentation spécifique au sexe du taux de protéines urinaires et du rapport albumine-créatinine a été observée chez les hommes.

**Conclusions:**

Les tendances observées pour les paramètres ETT et biochimiques mettent en évidence la nature graduelle et insidieuse de la progression de la MF, et soulignent l'importance de prendre en compte différents critères d'évaluation multiparamétriques, y compris la DLG, la fonction auriculaire et des biomarqueurs, lors de l'évaluation de l'issue de la MF.

Fabry disease (FD) is an X-linked inherited lysosomal storage disorder caused by deficiency in the enzyme α-galactosidase A,^1^ leading to progressive, multiorgan accumulation of sphingolipid, namely globotriaosylceramide (Gb3).^2^ Myocardial Gb3 accumulation begins in childhood, manifesting in adulthood primarily by triggering left ventricular hypertrophy (LVH).^3^^,^^4^ Cardiac involvement also is characterized by myocardial fibrosis that is associated with alteration in global longitudinal strain (GLS), a powerful focus for arrhythmia.^5^ A 3-phase development process of FD cardiomyopathy has been proposed, with sphingolipid accumulation, myocyte hypertrophy, and inflammation, leading to myocardial fibrosis and impairment.^6^ These cross-sectional data suggested differences between biological sexes in the development of cardiomyopathy during the myocyte hypertrophy and inflammation phase; more severe hypertrophy develops in male patients, and the onset of fibrosis precedes hypertrophy in female patients.^7^ These findings require confirmation with longitudinal assessment.

Transthoracic echocardiograms (TTEs) are the first-line modality to investigate cardiac involvement in FD, and they are recommended for regular, routine review.^8^ Tissue Doppler imaging (TDI) and speckle tracking of GLS hold promise in the detection of subclinical cardiomyopathy, prior to LVH, and impairments in GLS have been shown to reflect regional fibrosis of the myocardium on late gadolinium enhancement using cardiac magnetic resonance imaging (CMR).^9^ Speckle tracking strain is also a sensitive method of identifying right ventricular (RV) involvement,^10^ whereas altered left atrial (LA) strain may be associated with worsening diastolic function, exercise incapacity, and adverse clinical events, including atrial fibrillation (AF).^11^

Analyses of longitudinal trends in atrial and ventricular changes in FD are currently lacking in the literature. Given this context, data were retrospectively collected and analyzed from a cohort of patients with FD undergoing routine follow-up care. The primary aim of this study was to quantify trends in TTE parameters, linked to relevant physiological and biochemical markers, with the hypothesis that these may be more sensitive at detecting changes and, therefore, may serve as better targets for therapy in FD.

## Methods

### Study population

This observational, retrospective study included adults with FD attending a multidisciplinary clinic in the Centre for Rare Diseases at the Queen Elizabeth Hospital, Birmingham, United Kingdom (QEHB). The clinic incorporates cardiology, metabolic treatment, respiratory treatment, physiotherapy, and dietetics. FD diagnosis was based on plasma and leucocyte α-galactosidase A enzyme activity and confirmed by galactosidase-α (*GLA*) gene sequencing. Records relating to all clinic visits between November 2011 (the introduction of the multidisciplinary FD clinic) and March 2023 were interrogated, to identify all patients receiving follow-up care during this period. The first TTE performed at the centre was used to define the time point of entry to this study. Patients attended the clinic for follow-up assessments, which included clinical examination, TTE, and biochemical measurements, including relevant cardiac biomarkers. Although the clinic was established to provide face-to-face review, the frequency of outpatient attendance changed following the 2019 coronavirus (COVID-19) pandemic, and many visits were switched to online or telephone communication.

### TTE

The TTEs were performed by an accredited sonographer (A.M.A.) using IE33 and EPIC ultrasound systems (Phillips Electronics, Farnborough, United Kingdom), according to the British Society of Echocardiography minimum dataset.^12^^,^^13^ Diastolic function was assessed by an experienced imaging cardiologist (R.P.S.), according to existing guidelines.^12^^,^^14^ Linear internal measurements were obtained from 2-dimensional (2D) images in the parasternal long axis, measured immediately below the mitral valve (MV) leaflet tips, with left ventricular (LV) mass calculated using the Devereux formula^15^; 2D volumetric measurements were performed using the biplane summation of discs method, with separate acquisitions optimized for left atrial volumetric measurement. Apical 4-chamber 2D acquisitions were made at the time of examination for quantification of speckle-tracking strain in each case, following visual assessment of appropriate segmental tracking quality. This assessment was performed using QLAB Cardiac Analysis (Phillips Electronics). Measures of LA strain were not recorded as part of routine patient follow up care. Given this, the apical 2-chamber 2D views were assessed retrospectively, when available (by A.M.A. and A.R.), using TomTec off-cart software (Phillips Electronics). Study authors were not blinded to the demographic data or time of the study. A full list of the TTE parameters included is reported in Supplemental Table S1. Reproducibility was assessed for 4 of the LA strain parameters using intraclass correlation coefficients.^16^ Two observers assessed data for N = 10 patients, and achieved intraclass correlation coefficients of 0.97-1.00, indicating excellent inter-rater consistency (Supplemental Table S2). For patients in AF, an average of the measurements taken over 5 cycles was calculated.

### Data extraction

For each patient visit, electronic records were reviewed to extract clinical, physiological, and biochemical data. In cases in which blood samples were not collected on the day of the visit, results from the time point closest to this date, within a maximum of ± 90 days, were used. If levels were reported as being below a lower threshold, a value 1 significant figure lower than the threshold was assumed (eg, a value of 4 ng/L for Troponin of “ < 5 ng/L”); this usage impacted measurements for the albumin:creatinine ratio (ACR) (< 2.3 mg/mmol; 40% of measurements), the Troponin level (< 5 ng/L; 21%), and the urine protein level (< 0.005 mg/dL; 21%). Troponin levels were quantified using the Troponin-T level at the beginning of the study period. The assay used was then changed in 2019, with Troponin-I used subsequently. Given this method, the type of Troponin test used was recorded when extracting the data, so that Troponin-T and Troponin-I measurements could be analyzed separately.

Regarding comorbidities, ischemic heart disease was defined as previous myocardial infarction or prior coronary revascularization. Diabetes mellitus included a history of type 1 or type 2 diabetes mellitus, regardless of whether treatment was being received currently. Chronic kidney disease (CKD) was defined as an estimated glomerular filtration rate (eGFR; using the CKD Epidemiology Collaboration 2021 equation^17^) < 90 mL/min per 1.73 m^2^, and was divided into stages 1-5.^16^ Hypertension was defined based on evidence of a formal diagnosis and the initiation of treatment.

### Ethics

The study was approved by the West Midlands South Birmingham Research Ethics Committee (23/WM/0180 IRAS 325613). The study was conducted in accordance with local legislation and institutional requirements. The ethics committee waived the requirement of written informed consent for participation from the participants, as the study used routinely collected clinical data acquired from a research database.

### Statistical analysis

Initially, patients with data for serial visits were compared to those with data from only a single visit, using Mann–Whitney *U* tests for ordinal or continuous variables, and Fisher’s exact tests for categorical variables. Longitudinal trends in TTE parameters and physiological or biochemical markers were then assessed for the cohort of patients who attended at least 2 visits during the study period. This assessment was achieved using generalized estimating equation (GEE) models to estimate the trends over time for the cohort, while accounting for the non-independence of repeated measures on the same patient. Further details about the rationale for using this approach, as well as information about the models used, are reported in Supplemental Appendix S1. However, to summarize, the models fitted a trendline to the visit-level data, the gradient of which represented the estimated rate of change over time for the cohort. For parameters that were found to have linear trends over time, these gradients represented the number of units of increase per year. For parameters for which the units were percentages, such as LV ejection fraction (LVEF), gradients represented percentage point (pp) increases per year; for example, a gradient of 10 pp per year represents an increase from 40% to 50% in 1 year. For parameters with log-linear trends, gradients were reported as percentage increases per year; for example, a gradient of 10% per year represents an increase from 40% to 44% in 1 year.

The GEE models were then extended to include sex and an interaction term as additional factors. These models estimated the gradients for male and female patients separately, with the *P*-value for the interaction term representing a comparison between these gradients. Subgroup analyses were additionally performed based on whether patients were on disease-modifying FD therapy (DMT)—that is, enzyme replacement therapy (ERT) or oral chaperone therapy—at the first visit.

Finally, changes over time in selected TTE parameters or blood markers were compared, to identify correlations between the observed trends. Separate regression models were produced for each patient, the gradients of which were used as an estimate of their rate of change. Correlations of this patient-level data between parameters were then quantified using Spearman’s rank correlation coefficients (rho).

All analyses were performed using SPSS 24 (IBM, Armonk, NY), with *P* < 0.05 deemed to be indicative of statistical significance throughout. Continuous variables are reported as mean ± standard deviation (SD) when they were approximately normally distributed, with non-normal variables reported as median (interquartile range [IQR]), unless stated otherwise. Missing data were addressed using pairwise deletion; specifically, visits for which data were unavailable for some TTE parameters or physiological or biochemical markers were excluded from only the analyses of the affected parameters or markers.

## Results

### Cohort characteristics

**Figure 1:**
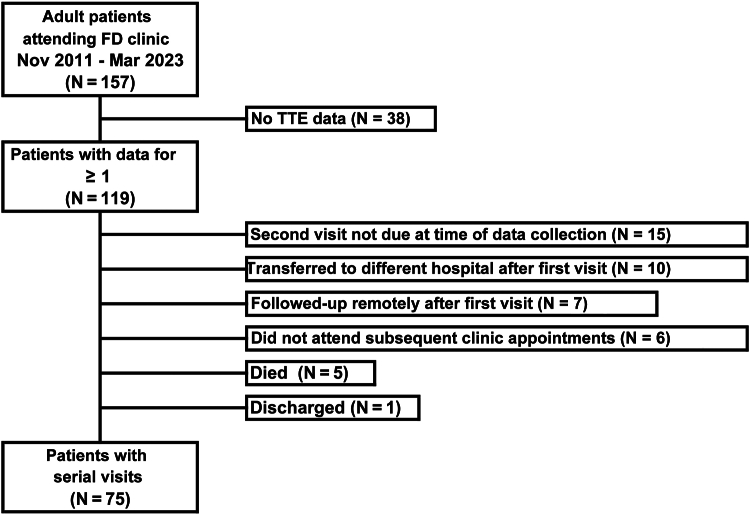
Study flowchart. FD, Fabry disease; Mar, March; Nov, November; TTE, transthoracic electrocardiogram.

A total of 157 patients attended the FD clinic between November 2011 and March 2023, of whom 119 had data available for at least one TTE. Of these, 75 patients had data for serial visits from which longitudinal trends could be estimated, with the remaining 44 attending only a single visit; the reasons for this are reported in Figure 1. Comparisons between these 2 groups revealed no significant differences in patient demographics, comorbidities, or physiological or biochemical markers (Table 1). However, patients with serial visits were significantly more likely than those with only a single visit to have classical FD mutations, to be on ERT at the first visit, to have significantly higher LV maximum wall thickness and LVEF, and to have more negative LA global circumferential strain (Table 2).

**Table 1:** Cohort characteristics at first visit

Characteristic	Cohort				*P*
	Serial visits		Single visit only		
	N	Statistic	N	Statistic	
Total number of visits	75	4 (2, 5)	—	—	—
Time from first to last visit, mo	75	55 (28, 82)	—	—	—
Age, y	75	50 (37, 59)	44	49 (33, 62)	0.943
Sex, % female	75	40 (53)	44	27 (61)	0.447
Mutation, % classical	75	43 (57)	44	16 (36)	**0.037**
Body surface area, m^2^	74	1.85 ± 0.24	44	1.85 ± 0.27	0.889
**Comorbidities**					
Diabetes mellitus	75	5 (7)	43	2 (5)	1.000
Hypertension	75	17 (23)	43	10 (23)	1.000
Hypercholesterolemia	75	20 (27)	43	8 (19)	0.374
Ischemic heart disease	75	5 (7)	43	4 (9)	0.722
Stroke	75	3 (4)	43	4 (9)	0.256
Angiokeratoma	75	7 (9)	43	4 (9)	1.000
Chronic kidney disease	73		40		0.324†
No		25 (34)		17 (41)	
Stage					
1		16 (22)		8 (20)	
2		22 (30)		12 (29)	
3a		3 (4)		1 (2)	
3b		2 (3)		0 (0)	
4		2 (3)		2 (5)	
5		3 (4)		0 (0)	
Unknown∗		—		1 (2)	
**Physiological and biochemical markers**					
eGFR, mL/min per 1.73 m^2^	71	89 ± 31	40	95 ± 29	0.450
Hemoglobin, g/L	60	137 ± 15	36	137 ± 13	0.880
Cholesterol, mmol/L	67	4.7 ± 1.0	37	4.9 ± 1.4	0.674
Troponin-I, ng/L	15	21 (4, 81)	23	4 (4, 36)	0.052
Troponin-T, ng/L	31	10 (4, 37)	13	14 (9, 24)	0.525
NT-proBNP, ng/L	59	144 (58, 1066)	35	144 (60, 702)	0.757
ACR, mg/mmol	66	2.5 (2.2, 15.2)	38	2.2 (2.2, 8.4)	0.302
Urine protein, mg/dL	66	1.7 (0.4, 9.3)	38	1.4 (0.5, 5.4)	0.570
Heart rate, bpm	75	64 ± 13	44	70 ± 16	0.110
Systolic BP, mm Hg	75	132 ± 17	44	133 ± 16	0.668
Diastolic BP, mm Hg	75	77 ± 10	44	78 ± 10	0.858
**Medication**					
Statins	75	23 (31)	44	15 (34)	0.839
ACE-i	75	30 (40)	44	15 (34)	0.562
Disease-modifying therapy	75		44		**0.001**
None		23 (31)		21 (48)	
ERT		43 (57)		10 (23)	
OCT		9 (12)		13 (30)	

Continuous variables are reported as median (interquartile range), or as mean ± standard deviation, as appropriate, with *P*-values from Mann–Whitney *U* tests. Categorical variables are reported as n (%), with *P*-values from Fisher’s exact tests, unless stated otherwise. Bold *P*-values are significant at *P* < 0.05.

ACE-i, angiotensin-converting enzyme inhibitor; ACR, albumin:creatinine ratio; BP, blood pressure; bpm, beats per minute; eGFR, estimated glomerular filtration rate; ERT, enzyme replacement therapy; NT-proBNP, N-terminal-pro-B-type natriuretic peptide; OCT, oral chaperone therapy.

∗ One patient was known to have chronic kidney disease, but had no stage recorded; this case was excluded when calculating the *P*-value.

† *P*-value is from the Mann–Whitney *U* test, as the factor is ordinal.

**Table 2:** Transthoracic echocardiogram (TTE) parameters at first visit

Parameter	Cohort				*P*
	Serial visits		Single visit only		
	N	Statistic	N	Statistic	
**Ventricular dimensions, volume, and function**					
LVIVSd, cm	74	1.3 (1.0, 1.6)	44	1.1 (0.9, 1.5)	0.054
LVEDd, cm	74	4.4 ± 0.4	44	4.5 ± 0.7	0.659
LVPWd, cm	74	1.2 ± 0.3	44	1.1 ± 0.3	0.156
LVESd, cm	67	2.9 ± 0.4	42	3.0 ± 0.6	0.413
LVEDvol 2D, mL	54	87 ± 27	36	92 ± 30	0.458
LVESvol 2D, mL	54	32 (25, 40)	36	33 (27, 44)	0.468
LVEF-BP, %	53	63 ± 5	37	62 ± 7	0.336
MAPSE, cm	65	14 ± 4	36	13 ± 3	0.743
TAPSE, cm	66	22 ± 4	40	22 ± 5	0.865
LVM, g	74	196 (142, 292)	44	173 (125, 254)	0.118
LVMi, g/m^2^	73	104 (80, 153)	44	94 (73, 127)	0.111
LV MWT, cm	74	1.4 ± 0.4	44	1.2 ± 0.4	**0.049**
GLS A4C, %	31	–17.6 ± 3.3	29	–17.5 ± 4.1	0.905
**Doppler studies and ratios**					
Tissue Doppler imaging, cm/s					
Lat s	74	9 ± 3	43	9 ± 3	0.672
Lat e	74	11 ± 5	43	12 ± 6	0.261
Lat a	69	8 ± 2	41	8 ± 2	0.488
Sep s	73	8 ± 2	42	8 ± 2	0.923
Sep e	72	8 ± 4	43	9 ± 4	0.487
Sep a	68	8 ± 2	41	7 ± 2	0.065
RV s	57	13 ± 3	36	13 ± 2	0.766
RV e	56	11 ± 4	34	12 ± 4	0.352
RV a	53	12 ± 3	33	12 ± 4	0.838
heading MV E max, m/s	73	80 ± 20	43	82 ± 22	0.562
MV A max, m/s	70	65 ± 19	41	65 ± 20	0.795
E/A	70	1.34 ± 0.56	41	1.41 ± 0.69	0.840
AV Vmax, m/s	71	131 (118, 150)	42	129 (114, 141)	0.282
LVOT Vmax, m/s	69	106 (93, 120)	44	101 (91, 116)	0.297
TV E, m/s	61	55 ± 15	38	58 ± 15	0.095
TV A, m/s	56	43 ± 12	36	44 ± 14	0.933
TR Vmax, cm/s	35	214 ± 34	16	229 ± 38	0.108
**LA dimensions and function**					
V, mL	69	43 (33, 60)	43	42 (35, 52)	0.548
Vi, mL/m^2^	63	23 (18, 31)	42	21 (19, 28)	0.521
GCS, %	57	–33 (–50, –14)	37	–17 (–35, –11)	**0.011**
GLS, %	57	–28 (–38, –15)	37	–23 (–32, –15)	0.138
EF, %	57	51 ± 20	37	42 ± 17	**0.029**
FAC, %	57	39 ± 17	37	32 ± 12	0.071

Data are reported as median (interquartile range) or mean ± standard deviation, as appropriate, with *P*-values from Mann–Whitney *U* tests. Bold *P*-values are significant at *P* < 0.05.

a, annulus; AV, atrioventricular; A4C, apical 4-chamber; E, E wave; e, early; EDd, end diastolic diameter; EDvol, end diastolic volume; EF, ejection fraction; ESd, end systolic diameter; ESvol, end systolic volume; FAC, fractional area change; GCS, global circumferential strain GLS, global longitudinal strain; IVSd, interventricular septal diameter; LA, left atrial; Lat, lateral; LV, left ventricular; MAPSE, mitral annular plane systolic excursion; M, mass; max, maximum; Mi, mass index; MV, mitral valve; MWT, maximum wall thickness; OT, outflow tract; PWd, P-wave dispersion; RV, right ventricular; S, septal; s, systolic; TAPSE, tricuspid annular plane systolic excursion; TR, tricuspid regurgitation; TV, triscuspid valve; V, volume; Vi, volume index.

Subsequent analysis included only the 75 patients who had serial visits; these patients attended a total of 305 visits (median: 4 per patient; range: 2-10) over a median of 55 months (IQR: 28-82) of clinical follow-up care. At the first visit, the cohort had a median age of 50 years (IQR: 37-59), and 53% were female. Most patients were on DMT at the first visit (69%), with 40% on angiotensin-converting enzyme inhibitors (ACE-i); ACE-i usage was similar in male and female patients (43% vs 38%, *P* = 0.646).

### Trends in TTE parameters

**Figure 2:**
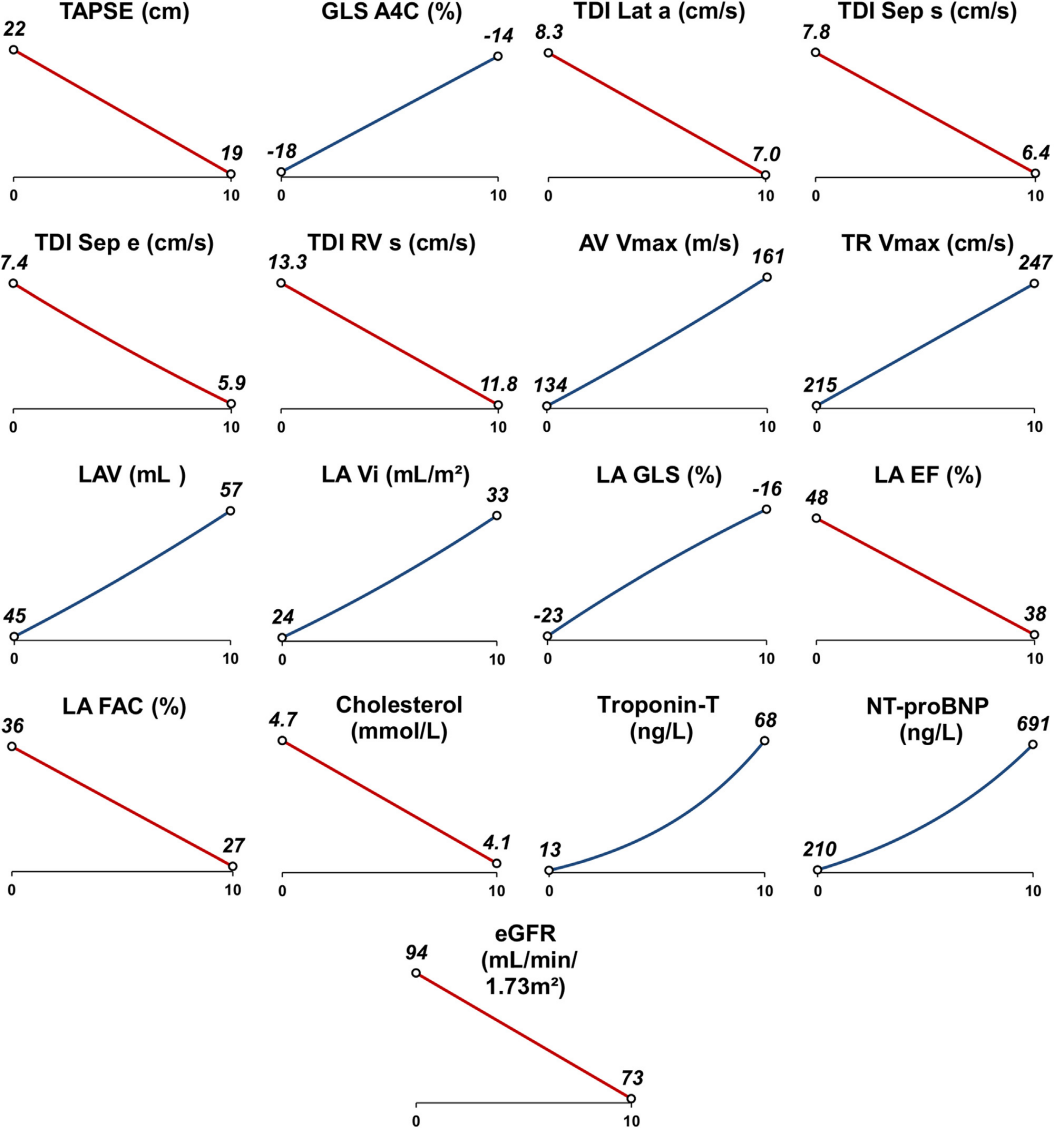
Significant longitudinal changes in transthoracic electrocardiogram (TTE) and biochemical parameters. Diagrams show the generalized estimating equation models in Tables 3 and 4 for parameters found to have significant trends over time. The x-axis represents the number of years from the first scan, with points representing the modelled levels at the time of the first scan, and after 10 years of follow-up care. Positive and negative trends are represented by **blue lines** and **red lines**, respectively. e, early; EF, ejection fraction; eGFR, estimated glomerular filtration rate; FAC, fractional area change; GLS, global longitudinal strain; LA, left atrial; Lat, lateral; NT-proBNP, N-terminal-pro-B-type natriuretic peptide; RV, right ventricular; s, systolic; Sep, septal; TAPSE, tricuspid annular plane systolic excursion; TDI, tissue Doppler imaging; TR, tricuspid regurgitation; Vi, index volume; Vmax, maximum velocity.

Analyses of longitudinal trends were then performed, with the resulting gradients reported in Table 3, and significant trends are shown in Figure 2. For example, tricuspid annular plane systolic excursion (TAPSE) was found to decrease significantly (*P* = 0.004), with an average change of –0.31 cm (95% confidence interval [CI]: –0.52, –0.10) per year (Table 3), which is equivalent to a reduction from 22 cm to 19 cm over a decade of follow-up evaluation (Fig. 2). The key findings are summarized below:

**Table 3:** Longitudinal trends in transthoracic echocardiogram (TTE) parameters

Parameter	Patients, N	Visits, N	Gradient per y (95% CI)	*P*
**Ventricular dimensions, volume, and function**				
LVIVSd, %∗	75	299	0.1 (–0.8, 1.0)	0.852
LVEDd, cm	75	299	0.01 (–0.02, 0.04)	0.436
LVPWd, cm	75	299	0.01 (0.00, 0.02)	0.162
LVESd, cm	75	283	0.00 (–0.02, 0.02)	0.825
LVEDvol 2D, %∗	73	236	1.1 (–0.3, 2.6)	0.126
LVESvol 2D, %∗	73	235	1.3 (–0.5, 3.1)	0.157
LVEF-BP, pp†	73	234	–0.21 (–0.47, 0.05)	0.111
MAPSE, %∗	75	232	–1.2 (–2.5, 0.0)	0.052
TAPSE, cm	75	278	–0.31 (–0.52, –0.10)	**0.004**
LVM, %∗	75	299	0.6 (–0.5, 1.7)	0.287
LVMi, %∗	75	296	0.8 (–0.4, 1.9)	0.197
LV MWT, cm	75	299	0.00 (–0.01, 0.01)	0.785
GLS A4C, pp†^,^‡	69	174	0.37 (0.12, 0.63)	**0.004**
**Doppler studies and ratios**				
Tissue Doppler imaging				
Lat s, cm/s	75	290	–0.11 (–0.22, 0.01)	0.064
Lat e, %∗	75	289	–1.3 (–3.0, 0.3)	0.115
Lat a, cm/s	73	264	–0.13 (–0.23, –0.02)	**0.017**
Sep s, cm/s	75	282	–0.13 (–0.20, –0.06)	**< 0.001**
Sep e, %∗	74	278	–2.2 (–3.7, –0.7)	**0.003**
Sep a, cm/s	71	252	–0.07 (–0.16, 0.02)	0.153
RV s, cm/s	75	236	–0.15 (–0.29, 0.00)	**0.046**
RV e, cm/s	74	229	–0.08 (–0.27, 0.11)	0.394
RV a, cm/s	70	212	–0.11 (–0.34, 0.12)	0.339
Heading				
MV E max, m/s	75	296	–0.03 (–0.66, 0.61)	0.937
MV A max, m/s	71	268	0.81 (–0.27, 1.88)	0.140
E/A, %∗	71	268	–0.9 (–2.7, 1.0)	0.358
AV Vmax, %∗	75	293	1.9 (0.6, 3.1)	**0.003**
LVOT Vmax, %∗	75	288	0.0 (–1.2, 1.1)	0.939
TV E, m/s	74	238	–0.23 (–0.87, 0.42)	0.487
TV A, m/s	71	213	0.46 (–0.31, 1.23)	0.246
TR Vmax, cm/s	56	147	3.2 (0.9, 5.5)	**0.007**
**Left atrial dimensions and function**				
V, %∗	75	287	2.4 (0.3, 4.5)	**0.022**
Vi, %∗	75	268	3.4 (1.5, 5.4)	**< 0.001**
GCS, %∗^,^‡^,^§	74	238	3.2 (–0.9, 7.1)	0.127
GLS, %∗^,^‡^,^§	74	238	3.4 (0.7, 6.0)	**0.014**
EF, pp†	74	238	–0.99 (–1.80, –0.17)	**0.018**
FAC, pp†	74	236	–0.83 (–1.41, –0.26)	**0.005**

All parameter values are per year. Results are from generalized estimating equation models on patients with ≥ 2 visits (N=75), with the stated TTE parameter as the dependent variable, and the timing of the visit as a covariate. Gradients represent the yearly increases in the stated parameter, and they are reported alongside 95% confidence intervals (CIs). Bold *P*-values are significant at *P* < 0.05.

a, annulus; AV, atrioventricular; A4C, apical 4-chamber; E, E wave; e, early; EDd, end diastolic diameter; EDvol, end diastolic volume; EF, ejection fraction; ESd, end systolic diameter; ESvol, end systolic volume; FAC, fractional area change; IVSd, interventricular septal diameter; LA, left atrial; Lat, lateral; LV, left ventricular; MAPSE, mitral annular plane systolic excursion; M, mass; max, maximum; Mi, mass index; MV, mitral valve; MWT, maximum wall thickness; OT, outflow tract; PWd, P-wave dispersion; RV, right ventricular; S, septal; s, systolic; TAPSE, tricuspid annular plane systolic excursion; TR, tricuspid regurgitation; TV, triscuspid valve; V, volume; Vi, volume index; 2D, 2-dimensional.

∗ Values were log_2_-transformed prior to analysis, to improve model fit; the resulting gradients were then anti-logged, and converted to percentage increases per year.

† For TTE parameters that are measured as percentages, the gradients represent percentage point (pp) increases per year; for example, a gradient of 1 would represent an increase from 4% to 5% in 1 year.

‡ Global circumferential strain (GCS) and global longitudinal strain (GLS) values were recorded as negative percentages; hence, the positive gradient indicates a longitudinal trend toward zero.

§ To normalize the negatively skewed distribution, absolute values were taken, to convert values from negative to positive, which were subsequently log_2_-transformed; the direction of the resulting gradient was then reversed to reflect the original scale.

Ventricular dimensions did not change significantly over time. Also showing no significant change were LV mass, indexed LV mass, and LVEF. However, significant increasing impairments over time were observed for TAPSE and GLS apical 4-chamber (A4C). Significant changes were also observed on TDI, with septal s’, septal e’, lateral a’, and RV s’ all decreasing over time.

Biplane LA volume (including indexed) increased significantly over time, with LA GLS becoming significantly more impaired, and LA ejection fraction (EF) and LA fractional area change both decreasing over time.

In patients with tricuspid regurgitation (TR), for whom a measurable trace could be obtained (N = 7 excluded with incomplete TR Doppler signal), the TR maximum velocity (Vmax) increased significantly over time. No clinically significant changes were noted in the MV and aortic valve forward velocities or regurgitation.

### Trends in physiological and biochemical markers

Analyses of physiological and biochemical markers are reported in Table 4, with significant trends shown in Figure 2. These analyses found significant increases over time in N-terminal-pro-B-type natriuretic peptide (NT-proBNP) and Troponin-T levels, with no associated changes in blood pressure. Initial analysis of eGFR produced a poor-fitting model, due to the high values in those with advanced CKD being extreme outliers. Because of this poor fit, the 5 patients with stage 4-5 CKD were excluded from the analysis, to produce a reliable model for the remainder of the cohort. This model showed that eGFR decreased significantly over time. A significant reduction over time in cholesterol levels also was observed. Given that NT-proBNP increased significantly over time, a post hoc decision was made to identify other TTE parameters that changed in parallel with NT-proBNP level. This analysis found that increases in NT-proBNP level were significantly correlated with the following: increases in LV mass, indexed LV mass, LV GLS A4C, and LA global circumferential strain; and a reduction in LVEF (Supplemental Table S3; Supplemental Fig. S1).

**Table 4:** Longitudinal trends in physiological and biochemical markers

Marker	Patients, N	Visits, N	Gradient per y (95% CI)	*P*
Hemoglobin, g/L	74	226	–0.58 (–1.38, 0.23)	0.160
eGFR, mL/min per 1.73 m^2^∗	69∗	255∗	–2.1 (–3.0, –1, 3)	**< 0.001**
Troponin-I, %†	64	115	3.6 (–5.9, 14.1)	0.471
Troponin-T, %†	56	101	18.0 (8.2, 28.7)	**< 0.001**
NT-proBNP, %†	73	237	12.6 (5.5, 20.2)	**< 0.001**
ACR%†	73	239	3.9 (–1.3, 9.5)	0.145
Urine protein, %†	71	234	2.5 (–4.3, 9.8)	0.484
Cholesterol, mmol/L	74	258	–0.06 (–0.10, –0.02)	**0.007**
Systolic BP, mm Hg	75	305	–0.46 (–1.00, 0.08)	0.096
Diastolic BP, mm Hg	75	305	0.15 (–0.27, 0.57)	0.480

All marker values are per year. Results are from generalized estimating equation models on patients with ≥ 2 visits (N=75), with the stated physiological or biochemical marker as the dependent variable, and the timing of the visit as a covariate. Gradients represent the yearly increases in the stated parameter, and they are reported alongside 95% confidence intervals (CIs). Bold *P*-values are significant at *P* < 0.05.

ACR, albumin:creatinine ratio; BP, blood pressure; eGFR, estimated glomerular filtration rate; NT-proBNP, N-terminal-pro-B-type natriuretic peptide.

∗ Analysis of estimated glomerular filtration rate (eGFR) excluded patients with chronic kidney disease stage 4 or 5 at the first visit (N = 5), to improve model fit.

† Values were log_2_-transformed prior to analysis, to improve model fit; the resulting gradients were then anti-logged, and converted to percentage increases per year.

### Subgroup analysis by sex

Longitudinal trends in TTE parameters and physiological or biochemical markers were then quantified separately for male and female patients (Supplemental Table S4, a and /b). This analysis found significant interaction effects for LV end-diastolic dimension, 2D LV end-diastolic volume, and MV E max, all of which had greater increases over time in malevs vs female patients. Indexed LV mass also was found to have significant increases over time in male but not female patients, and TDI Lateral e’ showed a significant decrease over time in female patients. Of the biochemical markers considered, a significant interaction effect was observed for hemoglobin, which was found to be decreasing over time in male but not female patients, whereas significant increases in ACR and urine protein were found in male but not female patients.

### Subgroup analysis by DMT

Analyses also were performed after splitting patients into subgroups based on whether they were receiving DMT at the time of the first visit. However, given that disease severity is one of the factors considered when deciding to initiate a patient on DMT, these groups were subject to considerable selection bias (see Supplemental Table S5, a and b). For example, patients receiving DMT were much older at the first visit (median age, 52 vs 37 years, *P* = 0.001), had significantly more deranged biomarkers (eg, median NT-proBNP level, 567 vs 58 ng/L, *P* < 0.001), and more advanced disease on TTE (eg, median LV mass index, 134 vs 80 g/m^2^). Consequently, analyses of the trends within these subgroups (reported in Supplemental Table S6, a and b) likely are subject to considerable confounding, and hence will be of questionable reliability. Given this fact, the findings of this subgroup analysis must be interpreted cautiously. Notable changes include that of a reduction in systolic or diastolic LV dimensions, E/A ratio, mitral annular plane systolic excursion, and LV TDI Lat e and s in the non-DMT group, indicating potential LVH with worsening of diastolic and radial LV function. Increasing trends in NT-proBNP and Troponin-T level were largely confined to the DMT group, likely reflecting the advanced nature of cardiomyopathy in this subgroup.

## Discussion

This study is the first of its kind to demonstrate atrial and ventricular changes on echocardiography in FD, incorporating hematological, physiological, and biochemical markers. Over a median of 55 months, a significant, progressive decline occurred in longitudinal ventricular contraction, as measured by a decline in septal tissue velocity, impairment of LV GLS, a decrease in TAPSE, and a reduction in RV systolic tissue velocity. In conjunction, significant, progressive LA dilatation and impaired LA function occurred. These complementary changes in ventricular and atrial function were associated with increased pulmonary artery pressure, as measured by maximal tricuspid regurgitant velocity. These trends occurred in the absence of significant changes in systolic and diastolic blood pressure over time. Significant increases over time in biomarkers were observed, specifically NT-proBNP and Troponin-T levels. Although LVH has been considered the defining feature of FD cardiomyopathy, and the main cardiac marker for decision-making regarding DMT and evaluation of therapeutic efficacy, this study suggests that other structural and functional markers of cardiac involvement may be better targets.

A widely accepted finding is that early treatment is critical to prevent progression to irreversible tissue damage and organ failure, certainly before the onset of fibrosis, and most likely before LVH occurs.^18^ Using echocardiographic changes as a continuum, a multiparameter approach that combines altered longitudinal deformation, elevated LV filling pressure, atrial enlargement, and dysfunction may be a better endpoint for future studies into the effect of early initiation of DMT.

The RV is frequently involved in FD cardiomyopathy, with RV hypertrophy often seen in conjunction with LVH, but rarely resulting in impaired RV function.^19^ We demonstrate a significant, albeit small, reduction in TAPSE which, unsurprisingly, occurred alongside a reduction in RV s’. TAPSE impairment has been suggested to be an important prognostic imaging marker in FD.^20^ In keeping with this approach, we also demonstrated a longitudinal increase in TR Vmax, suggesting an increase in pulmonary pressure. RV involvement may be a primary manifestation but also could be secondary to an increase in afterload from pulmonary venous hypertension that often confers an adverse outcome in other cardiomyopathies.^21^^,^^22^ Further data acquisition on the timing and importance of RV function In FD is warranted.

A total of 13% of FD patients suffer with ischemic stroke or transient ischemic attack.^23^^,^^24^ This finding may be due to the direct effects of cerebral sphingolipid accumulation.^25^ However, cardioembolic stroke secondary to undiagnosed AF is likely to contribute, given the high prevalence of AF in FD.^26^ Atrial dysfunction can predict new onset and recurrence of AF in the general population, with similar findings in FD.^11^^,^^27^ A cross-sectional study found that LA reservoir strain was impaired in those without other evidence of cardiomyopathy, was worse in those with LVH, and was still worse in those with late enhancement.^28^ An earlier cross-sectional study found that LA dilatation and reduced atrial compliance occurred irrespective of the presence of LVH.^29^ Interestingly, many of these patients did not have LVH, suggesting that direct atrial glycosphingolipid accumulation was a contributory factor,^11^ a possibility that has been confirmed on histopathological studies in patients with FD.^4^ Our findings, from a larger patient cohort, confirmed gradual impairment of LA GLS, LA EF, and fractional area change over time, corresponding with increases in LA volume. Although atrial dilatation and altered compliance are an early sign of FD, our study shows that this condition is progressive.^28^ Targeting early increases in atrial volume and impairments in atrial function with closer monitoring strategies may be a worthwhile method of reducing the burden of stroke in FD, which occurs at a much younger age and at more than double the rate of that in the general population.^30^

### Limitations

Several limitations need to be considered when interpreting these findings. First, the retrospective and observational design of the study meant that patients had differing durations of follow-up periods, and variability in the time between visits, particularly during COVID-19, resulting in patients contributing different numbers of visits to the analysis. To account for this variability, the data were analyzed using a GEE approach, to adjust for within-patient correlations, and prevent the over-weighting of those patients who had more visits. However, the analysis still will have assigned a greater weight to these patients, which may introduce selection bias, if underlying differences were present between these patients and those that attended less frequently. Selection bias also may have been introduced by excluding those who had a single scan; these patients were found to have lower rates of classical mutations and to be less likely to be on ERT at the first visit.

Second, for Troponin, ACR, and urine protein, a considerable proportion of cases had levels that were below the limit of detection of the test. These patients were assigned a value equal to the test threshold, to allow for the measurements to be included in the analysis. However, use of this method means that patients with levels consistently below the limit of detection were assumed to have a gradient of zero, potentially resulting in an underestimate of the gradient for the cohort.

Third, analyses of Troponin were limited by the fact that 2 different tests were used, across the study period, that had to be analyzed separately, resulting in smaller sample sizes and shorter follow-up periods being included in each analysis. Fourth, for the comparisons of the trends in NT-proBNP level with changes in TTE parameters, the analysis required the gradients to be estimated individually for each patient. Consequently, these gradients were estimated based on a small number of data points, leading to a low level of precision, and inflated variability. This low level of precision, coupled with the relatively small sample size used when analyzing patient-level data, resulted in a low level of statistical power, meaning that only moderate-to-large effect sizes were detectable. Fifth, the sensitivity to detect changes in LV strain over time is impacted by the fact that apical 4-chamber views alone were used to acquire the LV GLS. Data collection started in 2011, when routine GLS assessment was limited to apical 4-chamber views only.

Finally, although use of this cohort to assess the effect of DMT on FD progression would have been interesting, such an analysis was not feasible in the present cohort. This lack of feasibility was due to the fact that disease severity was one of the factors considered when making the decision to commence DMT, which resulted in considerable selection bias. Consequently, patients receiving DMT were substantially older and had more advanced FD; these differences were too large to adjust for reliably, as a means to isolate the independent effect of therapy. Thus, although we have reported analyses for the DMT and non-DMT subgroups separately, for completeness, these analyses are subject to the aforementioned biases, and must be interpreted cautiously. However, a future study in this area is warranted, ideally with a prospective before-and-after study design. Future work should also incorporate serial ECG to assess for conduction abnormalities and multiparametric CMR with tissue characterization, due to its emerging importance in the detection and staging of FD cardiomyopathy.

### Conclusion

We demonstrate a progressive decline in LV and RV longitudinal contraction, an increase in atrial volume and impairment in atrial function, and an associated increase in maximal velocity of tricuspid regurgitant jet. These changes occurred against a backdrop of increased NT-proBNP over time, reflecting greater wall stress in the ventricle from elevated left ventricular end-diastolic pressure, resulting in higher pulmonary venous pressure. These processes were continuous, likely reflecting the insidious organ damage that takes place in FD due to sphingolipid deposition. As a result, these processes do not fit easily into a staged approach to prognosis, although the timing and rate of identified changes cannot be assessed accurately from these data. Understanding these changes and the time course over which they take place should be useful for planning future studies of novel agents to improve cardiovascular outcomes in FD. Although advances in electrocardiography and CMR will improve cardiomyopathy detection, TTE and biochemistry remain the reference method for investigating and staging FD cardiomyopathy, due their cost effectiveness, their noninvasiveness, and their ease of access.

## References

[1] Desnick R.J., Brady R., Barranger J. (2003). Fabry disease, an under-recognized multisystemic disorder: expert recommendations for diagnosis, management, and enzyme replacement therapy. Ann Intern Med.

[2] Sweeley C.C., Klionsky B. (1963). Fabry's disease: classification as a sphingolipidosis and partial characterization of a novel glycolipid. J Biol Chem.

[3] Elleder M., Elstein D., Altarescu G., Becm M. (2010). Fabry Disease.

[4] Sheppard M.N., Cane P., Florio R. (2010). A detailed pathologic examination of heart tissue from three older patients with Anderson-Fabry disease on enzyme replacement therapy. Cardiovasc Pathol.

[5] Krämer J., Niemann M., Störk S. (2014). Relation of burden of myocardial fibrosis to malignant ventricular arrhythmias and outcomes in Fabry disease. Am J Cardiol.

[6] Pieroni M., Moon J.C., Arbustini E. (2021). Cardiac involvement in Fabry disease: JACC review topic of the week. J Am Coll Cardiol.

[7] Niemann M., Herrmann S., Hu K. (2011). Differences in Fabry cardiomyopathy between female and male patients: consequences for diagnostic assessment. JACC Cardiovasc Imaging.

[8] Linhart A., Germain D.P., Olivotto I. (2020). An expert consensus document on the management of cardiovascular manifestations of Fabry disease. Eur J Heart Fail.

[9] Krämer J., Niemann M., Liu D. (2013). Two-dimensional speckle tracking as a non-invasive tool for identification of myocardial fibrosis in Fabry disease. Eur Heart J.

[10] Morris D.A., Blaschke D., Canaan-Kühl S. (2015). Global cardiac alterations detected by speckle-tracking echocardiography in Fabry disease: left ventricular, right ventricular, and left atrial dysfunction are common and linked to worse symptomatic status. Int J Cardiovasc Imaging.

[11] Pichette M., Serri K., Pagé M. (2017). Impaired left atrial function in Fabry disease: a longitudinal speckle-tracking echocardiography study. J Am Soc Echocardiogr.

[12] Robinson S., Rana B., Oxborough D. (2020). A practical guideline for performing a comprehensive transthoracic echocardiogram in adults: the British Society of Echocardiography minimum dataset. Echo Res Pract.

[13] Wharton G., Steeds R., Allen J. (2015). A minimum dataset for a standard adult transthoracic echocardiogram: a guideline protocol from the British Society of Echocardiography. Echo Res Pract.

[14] Nagueh S.F., Smiseth O.A., Appleton C.P. (2016). Recommendations for the evaluation of left ventricular diastolic function by echocardiography: an update from the American Society of Echocardiography and the European Association of Cardiovascular Imaging. Eur Heart J Cardiovasc Imaging.

[15] Devereux R.B., Reichek N. (1977). Echocardiographic determination of left ventricular mass in man. Anatomic validation of the method. Circulation.

[16] Levin A., Stevens P.E., Bilous R.W. (2013). Kidney disease: Improving global outcomes (KDIGO) CKD work group. KDIGO 2012 clinical practice guideline for the evaluation and management of chronic kidney disease. Kidney Int Suppl.

[17] Inker L.A., Eneanya N.D., Coresh J. (2021). New creatinine- and cystatin C-based equations to estimate GFR without race. N Engl J Med.

[18] Weidemann F., Niemann M., Breunig F. (2009). Long-term effects of enzyme replacement therapy on Fabry cardiomyopathy: evidence for a better outcome with early treatment. Circulation.

[19] Graziani F., Laurito M., Pieroni M. (2017). Right ventricular hypertrophy, systolic function, and disease severity in Anderson-Fabry disease: an echocardiographic study. J Am Soc Echocardiogr.

[20] Meucci M.C., Lillo R., Del Franco A. (2023). Prognostic implications of the extent of cardiac damage in patients with Fabry disease. J Am Coll Cardiol.

[21] Huntjens P.R., Zhang K.W., Soyama Y. (2021). Prognostic utility of echocardiographic atrial and ventricular strain imaging in patients with cardiac amyloidosis. JACC Cardiovasc Imaging.

[22] Hiemstra Y.L., Debonnaire P., Bootsma M. (2019). Prevalence and prognostic implications of right ventricular dysfunction in patients with hypertrophic cardiomyopathy. Am J Cardiol.

[23] Shi Q., Chen J., Pongmoragot J., Lanthier S., Saposnik G. (2014). Prevalence of Fabry disease in stroke patients--a systematic review and meta-analysis. J Stroke Cerebrovasc Dis.

[24] Mehta A., Ricci R., Widmer U. (2004). Fabry disease defined: baseline clinical manifestations of 366 patients in the Fabry Outcome Survey. Eur J Clin Invest.

[25] Politei J., Schenone A.B., Burlina A. (2014). Vertebrobasilar dolichoectasia in Fabry disease: the earliest marker of neurological involvement?. J Inborn Errors Metab Screening.

[26] Vijapurapu R., Roy A., Demetriades P. (2023). Systematic review of the incidence and clinical risk predictors of atrial fibrillation and permanent pacemaker implantation for bradycardia in Fabry disease. Open Heart.

[27] Hauser R., Nielsen A.B., Skaarup K.G. (2021). Left atrial strain predicts incident atrial fibrillation in the general population: the Copenhagen City Heart Study. Eur Heart J Cardiovasc Imaging.

[28] Halfmann M.C., Altmann S., Schoepf U.J. (2023). Left atrial strain correlates with severity of cardiac involvement in Anderson-Fabry disease. Eur Radiol.

[29] Boyd A.C., Lo Q., Devine K. (2013). Left atrial enlargement and reduced atrial compliance occurs early in Fabry cardiomyopathy. J Am Soc Echocardiogr.

[30] Sims K., Politei J., Banikazemi M., Lee P. (2009). Stroke in Fabry disease frequently occurs before diagnosis and in the absence of other clinical events: natural history data from the Fabry Registry. Stroke.

