# Supplementary material for: Utilization of Transthoracic Echocardiography and Biochemical Markers in Detecting Cardiomyopathy in Fabry Disease
Source: CJC Open. 2025 Jan 23;7(5):595–605. doi: 10.1016/j.cjco.2025.01.017 (PMC12105508; doi:10.1016/j.cjco.2025.01.017)

**SUPPLEMENTARY MATERIAL**

***Supplemental Appendix S1***

**STATISTICAL ANALYSIS**

Longitudinal trends in TTE parameters and physiological / biochemical markers were assessed for the cohort of patients who attended at least two visits during the study period. Since it would be expected that repeated measurements of a parameter on the same patient across multiple visits would be correlated, analysis of visit-level data would be subject to non-independence (i.e. such that two measurements made on the same patient would be more similar than measurements made on two different patients). If this were not accounted for, then the level of variability would likely be underestimated, and the analysis would also be excessively weighted towards patients with greater numbers of visits.

Consequently, analyses of trends were performed using generalised estimating equations (GEEs). These assumed an first-order autoregressive (AR[1]) correlation structure, with the within-subjects factor being the timing of the visit relative to the first visit, rounded to the nearest year. As such, the GEEs assumed that the correlation between repeated measurements on the same patient would be strongest when these were taken closer together (e.g. one year apart), and decline as visits were spaced further apart in time (e.g. several years apart). GEE models were produced with each TTE parameter and physiological / biochemical marker as the dependent variable, and the number of years (rounded to the nearest day) from the patient’s first visit during the study period to the present visit as a continuous covariate. As such, the models fitted a linear trendline to the visit-level data, the gradient of which was summarised as a number of units increase per year. For parameters where the units were percentages (e.g. LV ejection fraction), gradients represented percentage point (pp) changes per year; for example, a gradient of 10pp per year would represent an increase from 40% to 50% in one year.

The goodness-of-fit of the resulting models was then assessed graphically, by review of the residuals, to assess whether the assumption of a linear trend was reasonable. This was not the case for parameters that followed positively skewed distributions, for which the trends were better represented by a log-linear model. For these parameters, values were log_2_-transformed, before being set as the dependent variable in a GEE. To simplify the interpretation of the resulting models, the gradients were anti-logged and converted to percentage increases per year; for example, a gradient of 10% per year would represent an increase from 40% to 44% in a year. Negatively skewed distributions were observed for two parameters: LA GCS and LA GLS; since all values of these were negative, the absolute values were taken to reverse the direction of the skew, before applying a log_2_-transformation. The direction of the resulting gradient was then reversed, to undo the transformation.

The GEE models were also extended, to allow for comparisons of trends between subgroups of patients defined by biological sex. Specifically, two additional factors were added to the previously described models, namely sex, and the interaction between sex and the timing of the visit. As such, these models estimated the gradients for male and female patients separately, with the p-value for the interaction term representing a comparison between the gradients for males and females.

The changes over time in selected TTE parameters / biochemical markers were then compared, to identify correlations between the observed trends. This analysis required the trends over time to be estimated for each patient separately. As such, either linear or log-linear regression models were produced for each patient (as appropriate), with a TTE parameter / biochemical marker as the dependent variable, and the timing of the visit as a covariate. The gradients from the resulting models were then used to summarise the rate of change in the parameter for each patient. Correlations between these gradients were then quantified using Spearman’s rank correlation coefficients (rho); only those patients with data for more than two assessments for the pair of TTE parameters / biochemical markers being compared were included in this analysis, as gradients estimated from only two points would be unreliable. Where significant correlations were detected, regression models were produced to further visualise the associations between the trends.

***Supplemental Table S1 – TTE parameter definitions***

| **Parameter** | **Definition of Abbreviation** |
| --- | --- |
| **Ventricular Dimensions, Volume and Function** | |
| LVIVSd (cm) | Left ventricular intraventricular septal dimension |
| LVEDd (cm) | Left ventricular end-diastolic dimension |
| LVPWd (cm) | Left ventricular posterior wall dimension |
| LVESd (cm) | Left ventricular end-systolic dimension |
| LVEDvol 2D (ml) | Left ventricular end-diastolic volume - 2-dimension |
| LVESvol 2D (ml) | Left ventricular end-systolic volume - 2-dimension |
| LVEF-BP (%) | Left ventricular ejection fraction - Simpson's biplane |
| MAPSE (cm) | Mitral annular plane systolic excursion |
| TAPSE (cm) | Tricuspid annular plane systolic excursion |
| LVM (g) | Left ventricular mass (Devereux formula) |
| LVMi (g/m^2^) | Left ventricular mass indexed to body surface area |
| LV MWT (cm) | Left ventricular maximum wall thickness |
| GLS A4C (%) | Global longitudinal strain - apical 4-chamber |
| **Doppler Studies and Ratios** | |
| TDI Lat s (cm/s) | Lateral S wave |
| TDI Lat e (cm/s) | Lateral E wave |
| TDI Lat a (cm/s) | Lateral A wave |
| TDI Sep s (cm/s) | Septal S wave |
| TDI Sep e (cm/s) | Septal E wave |
| TDI Sep a (cm/s) | Septal A wave |
| TDI RV s (cm/s) | Right ventricular S wave |
| TDI RV e (cm/s) | Right ventricular E wave |
| TDI RV a (cm/s) | Right ventricular A wave |
| MV E max (m/s) | Mitral valve maximum E wave velocity |
| MV A max (m/s) | Mitral valve maximum A wave velocity |
| E/A | Mitral valve maximum E wave/A wave velocity |
| AV Vmax (m/s) | Aortic valve maximum velocity |
| LVOT Vmax (m/s) | Left ventricular outflow tract maximum velocity |
| TV E (m/s) | Tricuspid valve E wave |
| TV A (m/s) | Tricuspid valve A wave |
| TR Vmax (cm/s) | Tricuspid regurgitation maximum velocity |
| **Atrial Dimensions and Function** | |
| LAV (ml) | Left atrial volume |
| LA Vi (ml/m^2^) | Left atrial volume indexed to body surface area |
| LA GCS (%) | Left atrial global circumferential strain |
| LA GLS (%) | Left atrial global longitudinal strain |
| LA EF (%) | Left atrial ejection fraction |
| LA FAC (%) | Left atrial fractional area change |

***Supplemental Table S2 – Intraclass correlation coefficient for LA parameters***

| **Parameter** | ***Intraclass Correlation Coefficient (95% CI)*** |
| --- | --- |
| LA GCS | 0.999 (0.994-1.000) |
| LA GLS | 0.968 (0.883-0.992) |
| LA EF | 0.999 (0.995-1.000) |
| LA FAC | 0.999 (0.998-1.000) |

*Results are based on N=10 cases who were each assessed by two observers. Intraclass correlation coefficients are calculated using two-way mixed-effects models with absolute agreement and are reported for single measures. Definitions of the TTE parameter abbreviations are reported in* ***Supplemental Table S1****.*

***Supplemental Table S3 – Correlations between trends in NT-proBNP and trends in TTE parameters***

|  | **N** | **Rho** | **p-Value** |
| --- | --- | --- | --- |
| **Ventricular Dimensions, Volume and Function** | | | |
| LVIVSd **^a^** | 45 | 0.246 | 0.104 |
| LVEDd | 45 | 0.254 | 0.093 |
| LVPWd | 45 | 0.041 | 0.790 |
| LVESd | 45 | 0.133 | 0.382 |
| LVEDvol 2D **^a^** | 38 | 0.031 | 0.852 |
| LVESvol 2D **^a^** | 38 | 0.217 | 0.190 |
| LVEF-BP | 37 | -0.425 | **0.009** |
| MAPSE **^a^** | 40 | -0.284 | 0.075 |
| TAPSE | 45 | -0.118 | 0.438 |
| LVM **^a^** | 45 | 0.366 | **0.013** |
| LVMi **^a^** | 45 | 0.347 | **0.020** |
| LV MWT | 45 | 0.250 | 0.098 |
| GLS A4C **^b^** | 26 | 0.416 | **0.035** |
| **Doppler Studies and Ratios** | | | |
| TDI Lat s | 44 | 0.171 | 0.268 |
| TDI Lat e **^a^** | 44 | 0.066 | 0.669 |
| TDI Lat a | 41 | -0.145 | 0.366 |
| TDI Sep s | 43 | -0.111 | 0.480 |
| TDI Sep e **^a^** | 43 | 0.095 | 0.546 |
| TDI Sep a | 40 | -0.192 | 0.235 |
| TDI RV s | 42 | -0.164 | 0.300 |
| TDI RV e | 40 | 0.133 | 0.413 |
| TDI RV a | 38 | 0.195 | 0.240 |
| MV E max | 45 | 0.052 | 0.733 |
| MV A max | 43 | -0.293 | 0.056 |
| E/A **^a^** | 43 | 0.262 | 0.089 |
| AV Vmax **^a^** | 45 | 0.076 | 0.618 |
| LVOT Vmax **^a^** | 45 | -0.059 | 0.701 |
| TV E | 41 | 0.187 | 0.243 |
| TV A | 36 | 0.042 | 0.808 |
| TR Vmax | 21 | 0.208 | 0.366 |
| **Atrial Dimensions and Function** | | | |
| LAV **^a^** | 43 | -0.038 | 0.810 |
| LA Vi **^a^** | 42 | 0.005 | 0.977 |
| LA GCS **^a b c^** | 40 | 0.313 | **0.049** |
| LA GLS **^a b c^** | 40 | 0.298 | 0.062 |
| LA EF | 40 | -0.220 | 0.172 |
| LA FAC | 40 | -0.165 | 0.310 |

*Initially, linear regression models were produced for each patient separately, with the stated TTE parameter as the dependent variable, and the timing of the visit as a covariate. TTE parameters that were found to have non-linear trends over time in the analysis of overall trends in* ***Table 3*** *were log_2_-transformed prior to analysis, to improve model fit. The gradients from the resulting models were then used to summarise the rate of change in the TTE parameter for each patient. The rate of change in NT-proBNP for each patient was then estimated using the same approach. Spearman’s rho correlation coefficients were then used to compare the rate of change in NT-proBNP to that of each TTE parameter. These analyses included only those patients with data for more than two assessments of NT-proBNP and the TTE parameter being analysed, as gradients estimated from only two points would be unreliable. Bold p-values are significant at p<0.05. Definitions of the TTE parameter abbreviations are reported in* ***Supplemental Table S1****.* ***^a^*** *Values were log_2_-transformed prior to analysis, in order to improve model fit.* ***^b^*** *GCS and GLS values were recorded as negative percentages; hence, the positive gradient indicated a longitudinal trend towards zero. ^c^ To normalise the negatively skewed distribution, absolute values were taken, to convert values from negative to positive, which were subsequently log_2_-transformed; the direction of the resulting gradient was then reversed to reflect the original scale.*

***Supplemental Table S4a - Longitudinal trends in TTE parameters and physiological / biochemical markers by sex***

|  | **Female** | | **Male** | | **Interaction p-Value** |
| --- | --- | --- | --- | --- | --- |
|  | ***Gradient (95% CI)*** | ***p-Value*** | ***Gradient (95% CI)*** | ***p-Value*** |  |
| **Ventricular Dimensions, Volume and Function** | | | | | |
| LVIVSd (% per Year) **^a^** | 0.1% (-1.1%, 1.4%) | 0.823 | -0.3% (-1.3%, 0.7%) | 0.602 | 0.620 |
| LVEDd (cm per Year) | -0.02 (-0.05, 0.01) | 0.125 | 0.05 (0.00, 0.09) | **0.048** | **0.014** |
| LVPWd (cm per Year) | 0.01 (0.00, 0.03) | **0.013** | 0.00 (-0.01, 0.01) | 0.985 | 0.127 |
| LVESd (cm per Year) | -0.01 (-0.03, 0.02) | 0.659 | 0.00 (-0.03, 0.04) | 0.886 | 0.715 |
| LVEDvol 2D (% per Year) **^a^** | -0.6% (-2.1%, 1.0%) | 0.470 | 2.8% (0.6%, 5.0%) | **0.011** | **0.013** |
| LVESvol 2D (% per Year) **^a^** | 0.1% (-1.4%, 1.7%) | 0.850 | 2.5% (-0.3%, 5.4%) | 0.086 | 0.156 |
| LVEF-BP (pp per Year) **^b^** | -0.22 (-0.50, 0.05) | 0.112 | -0.19 (-0.60, 0.22) | 0.358 | 0.896 |
| MAPSE (% per Year) **^a^** | -1.7% (-3.5%, 0.0%) | 0.053 | -0.5% (-2.2%, 1.3%) | 0.582 | 0.326 |
| TAPSE (cm per Year) | -0.46 (-0.74, -0.17) | **0.002** | -0.16 (-0.46, 0.14) | 0.302 | 0.162 |
| LVM (% per Year) **^a^** | 0.3% (-1.2%, 1.8%) | 0.716 | 1.3% (-0.3%, 2.9%) | 0.106 | 0.357 |
| LVMi (% per Year) **^a^** | 0.0% (-1.6%, 1.5%) | 0.958 | 1.8% (0.3%, 3.3%) | **0.019** | 0.101 |
| LV MWT (cm per Year) | 0.01 (-0.01, 0.02) | 0.431 | -0.01 (-0.02, 0.01) | 0.499 | 0.300 |
| GLS A4C (pp per Year) **^b c^** | 0.20 (-0.08, 0.48) | 0.169 | 0.55 (0.25, 0.84) | **<0.001** | 0.092 |
| **Doppler Studies and Ratios** | | | | | |
| TDI Lat s (cm/s per Year) | -0.13 (-0.25, -0.02) | **0.025** | -0.08 (-0.27, 0.12) | 0.435 | 0.628 |
| TDI Lat e (% per Year) **^a^** | -3.2% (-5.2%, -1.1%) | **0.003** | 0.9% (-1.5%, 3.3%) | 0.467 | **0.010** |
| TDI Lat a (cm/s per Year) | -0.16 (-0.31, -0.01) | **0.032** | -0.10 (-0.25, 0.06) | 0.220 | 0.555 |
| TDI Sep s (cm/s per Year) | -0.16 (-0.24, -0.08) | **<0.001** | -0.09 (-0.20, 0.01) | 0.087 | 0.313 |
| TDI Sep e (% per Year) **^a^** | -3.3% (-5.1%, -1.5%) | **<0.001** | -1.1% (-3.4%, 1.3%) | 0.355 | 0.142 |
| TDI Sep a (cm/s per Year) | -0.07 (-0.19, 0.05) | 0.274 | -0.06 (-0.20, 0.07) | 0.361 | 0.948 |
| TDI RV s (cm/s per Year) | -0.24 (-0.41, -0.07) | **0.007** | -0.05 (-0.27, 0.17) | 0.649 | 0.188 |
| TDI RV e (cm/s per Year) | -0.16 (-0.40, 0.08) | 0.188 | 0.00 (-0.29, 0.29) | 0.992 | 0.405 |
| TDI RV a (cm/s per Year) | -0.20 (-0.53, 0.12) | 0.218 | -0.01 (-0.34, 0.31) | 0.930 | 0.419 |
| MV E max (m/s per Year) | -0.61 (-1.43, 0.22) | 0.148 | 0.68 (-0.22, 1.57) | 0.140 | **0.039** |
| MV A max (m/s per Year) | 0.56 (-0.42, 1.55) | 0.259 | 1.12 (-0.88, 3.13) | 0.272 | 0.624 |
| E/A (% per Year) **^a^** | -1.6% (-3.3%, 0.2%) | 0.082 | 0.0% (-3.4%, 3.5%) | 0.998 | 0.424 |
| AV Vmax (% per Year) **^a^** | 2.1% (0.6%, 3.6%) | **0.005** | 1.7% (-0.4%, 3.8%) | 0.119 | 0.743 |
| LVOT Vmax (% per Year) **^a^** | -0.1% (-1.0%, 0.9%) | 0.869 | 0.1% (-2.2%, 2.4%) | 0.952 | 0.904 |
| TV E (m/s per Year) | 0.03 (-0.94, 1.01) | 0.946 | -0.48 (-1.34, 0.38) | 0.270 | 0.436 |
| TV A (m/s per Year) | 0.74 (-0.31, 1.79) | 0.169 | 0.18 (-0.96, 1.32) | 0.752 | 0.484 |
| TR Vmax (cm/s per Year) | 1.4 (-1.9, 4.8) | 0.397 | 4.5 (1.2, 7.8) | **0.007** | 0.197 |

*Results are from generalised estimating equation models on patients with at least two visits (N=40 female; N=35 male), with the stated parameter as the dependent variable, and the timing of the visit, sex and an interaction term as covariates. The models were then evaluated to estimate the yearly gradients for each parameter for the male and female subgroups separately, which are reported alongside 95% confidence intervals (95% CIs). The p-value for the interaction term represents a comparison between these two gradients. Bold p-values are significant at p<0.05. Definitions of the TTE parameter abbreviations are reported in* ***Supplemental Table S1****. Analyses included only those TTEs for which the stated parameter was recorded – see* ***Tables 3 and 4*** *for the included sample sizes.* ***^a^*** *Values were log_2_-transformed prior to analysis, in order to improve model fit; the resulting gradients were then anti-logged, and converted to percentage increases per year.* ***^b^*** *For TTE parameters that are measured as percentages, the gradients represent percentage point (pp) increases per year; for example a gradient of 1 would represent an increase from 4% to 5% in one year.* **^c^** *GCS and GLS values were recorded as negative percentages; hence, the positive gradient indicates a longitudinal trend towards zero.*

***Supplemental Table S4b - Longitudinal trends in TTE parameters and physiological / biochemical markers by sex***

|  | **Female** | | **Male** | | **Interaction p-Value** |
| --- | --- | --- | --- | --- | --- |
|  | ***Gradient (95% CI)*** | ***p-Value*** | ***Gradient (95% CI)*** | ***p-Value*** |  |
| **Atrial Dimensions and Function** | | | | | |
| LAV (% per Year) **^a^** | 2.2% (-0.7%, 5.2%) | 0.135 | 2.9% (0.3%, 5.6%) | **0.032** | 0.753 |
| LA Vi (% per Year) **^a^** | 3.2% (0.3%, 6.1%) | **0.030** | 4.0% (1.6%, 6.4%) | **<0.001** | 0.680 |
| LA GCS (% per Year) **^a c d^** | 1.5% (-4.1%, 6.8%) | 0.589 | 4.9% (-2.0%, 11.4%) | 0.160 | 0.439 |
| LA GLS (% per Year) **^a c d^** | 5.5% (2.4%, 8.5%) | **<0.001** | 1.2% (-3.3%, 5.5%) | 0.597 | 0.111 |
| LA EF (pp per Year) **^b^** | -0.91 (-1.91, 0.10) | 0.076 | -1.09 (-2.55, 0.36) | 0.142 | 0.838 |
| LA FAC (pp per Year) **^b^** | -0.93 (-1.66, -0.21) | **0.012** | -0.73 (-1.72, 0.26) | 0.146 | 0.748 |
| **Physiological and Biochemical Markers** | | | | | |
| Haemoglobin (g/l per Year) | 0.34 (-0.61, 1.28) | 0.485 | -1.48 (-2.67, -0.29) | **0.015** | **0.019** |
| eGFR (ml/min/1.73m^2^ per Year) **^e^** | -1.9 (-2.9, -0.8) | **<0.001** | -2.4 (-3.8, -1.1) | **<0.001** | 0.523 |
| Troponin-I (% per Year) **^a^** | 4.3% (-12.5%, 24.2%) | 0.640 | 2.9% (-4.1%, 10.4%) | 0.420 | 0.894 |
| Troponin-T (% per Year) **^a^** | 20.4% (7.4%, 34.9%) | **0.001** | 12.9% (4.5%, 22.1%) | **0.002** | 0.365 |
| NT-proBNP (% per Year) **^a^** | 9.8% (4.2%, 15.8%) | **<0.001** | 14.8% (4.0%, 26.8%) | **0.006** | 0.441 |
| ACR (% per Year) **^a^** | -2.6% (-8.2%, 3.3%) | 0.378 | 12.8% (3.7%, 22.7%) | **0.005** | **0.005** |
| Urine Protein (% per Year) **^a^** | -5.7% (-15.3%, 5.0%) | 0.283 | 12.6% (3.1%, 23.1%) | **0.008** | **0.012** |
| Cholesterol (mmol/l per Year) | -0.02 (-0.07, 0.03) | 0.365 | -0.09 (-0.16, -0.03) | **0.005** | 0.101 |
| Systolic BP (mmHg per Year) | -0.45 (-1.17, 0.28) | 0.229 | -0.47 (-1.27, 0.34) | 0.256 | 0.970 |
| Diastolic BP (mmHg per Year) | 0.12 (-0.21, 0.45) | 0.464 | 0.18 (-0.59, 0.96) | 0.645 | 0.890 |

*Results are from generalised estimating equation models on patients with at least two visits (N=40 female, N=35 male), with the stated parameter as the dependent variable, and the timing of the visit, sex and an interaction term as covariates. The models were then evaluated to estimate the yearly gradients for each parameter for the male and female subgroups separately, which are reported alongside 95% confidence intervals (95% CIs). The p-value for the interaction term represents a comparison between these two gradients. Bold p-values are significant at p<0.05. Definitions of the TTE parameter abbreviations are reported in* ***Supplemental Table S1****. Analyses included only those for TTEs for which the stated parameter was recorded – see* ***Tables 3 and 4*** *for the included sample sizes.* ***^a^*** *Values were log_2_-transformed prior to analysis, in order to improve model fit; the resulting gradients were then anti-logged, and converted to percentage increases per year.* ***^b^*** *For TTE parameters that are measured as percentages, the gradients represent percentage point (pp) increases per year; for example a gradient of 1 would represent an increase from 4% to 5% in one year.* **^c^** *GCS and GLS values were recorded as negative percentages; hence, the positive gradient indicates a longitudinal trend towards zero. ^d^ To normalise the negatively skewed distribution, absolute values were taken, to convert values from negative to positive, which were subsequently log_2_-transformed; the direction of the resulting gradient was then reversed to reflect the original scale. ^e^* *Analysis of eGFR excluded patients with CKD stage 4 or 5 at the first visit (N=5), in order to improve model fit. ACR=Albumin:creatinine ratio, BP=Blood pressure, eGFR=Estimated glomerular filtration rate, NT-proBNP=N-terminal-pro-B-type natriuretic peptide.*

***Supplemental Table S5a – Cohort characteristics at first visit by disease-modifying therapy***

|  | **Disease-Modifying Therapy at First Visit** | | | | **p-**  **Value** |
| --- | --- | --- | --- | --- | --- |
|  | **No** | | **Yes** | |  |
|  | ***N*** | ***Statistic*** | ***N*** | ***Statistic*** |  |
| Total Number of Visits | 23 | 3 (2, 4) | 52 | 4 (3, 6) | 0.054 |
| First to Last Visit (Months) | 23 | 47 (27, 64) | 52 | 62 (29, 93) | 0.175 |
| Age (Years) | 23 | 37 (25, 53) | 52 | 52 (43, 61) | **0.001** |
| Sex (% Female) | 23 | 16 (70%) | 52 | 24 (46%) | 0.080 |
| Body Surface Area (m^2^) | 23 | 1.83 ± 0.22 | 51 | 1.86 ± 0.25 | 0.678 |
| **Comorbidities** | | | | | |
| Diabetes Mellitus | 23 | 1 (4%) | 52 | 4 (8%) | 1.000 |
| Hypertension | 23 | 2 (9%) | 52 | 15 (29%) | 0.074 |
| Hypercholesterolemia | 23 | 2 (9%) | 52 | 18 (35%) | **0.023** |
| Ischaemic Heart Disease | 23 | 0 (0%) | 52 | 5 (10%) | 0.315 |
| Stroke | 23 | 0 (0%) | 52 | 3 (6%) | 0.548 |
| Angiokeratoma | 23 | 0 (0%) | 52 | 7 (13%) | 0.093 |
| Chronic Kidney Disease | 22 |  | 51 |  | **0.009 ^a^** |
| *No* |  | 11 (50%) |  | 14 (27%) |  |
| *Stage 1* |  | 6 (27%) |  | 10 (20%) |  |
| *Stage 2* |  | 5 (23%) |  | 17 (33%) |  |
| *Stage 3a* |  | - |  | 3 (6%) |  |
| *Stage 3b* |  | - |  | 2 (4%) |  |
| *Stage 4* |  | - |  | 2 (4%) |  |
| *Stage 5* |  | - |  | 3 (6%) |  |
| **Physiological and Biochemical Markers** | | | | | |
| eGFR (ml/min/1.73m^2^) | 21 | 105 ± 17 | 50 | 82 ± 33 | **0.003** |
| Haemoglobin (g/l) | 18 | 132 ± 12 | 42 | 139 ± 15 | **0.022** |
| Cholesterol (mmol/l) | 19 | 4.4 ± 1.0 | 48 | 4.8 ± 1.0 | 0.237 |
| Troponin-I (ng/l) | 4 | 4 (4, 6) | 11 | 51 (18, 146) | **0.010** |
| Troponin-T (ng/l) | 12 | 4 (4, 10) | 19 | 26 (8, 46) | **0.005** |
| NT-proBNP (ng/l) | 19 | 58 (34, 85) | 40 | 567 (97, 1338) | **<0.001** |
| ACR (mg/mmol) | 21 | 2.2 (2.2, 3.0) | 45 | 3.1 (2.2, 21.2) | **0.015** |
| Urine Protein (mg/dl) | 21 | 0.8 (0.4, 1.9) | 45 | 2.7 (0.5, 11.9) | 0.051 |
| Heart Rate (bpm) | 23 | 64 ± 8 | 52 | 64 ± 15 | 0.704 |
| Systolic BP (mmHg) | 23 | 130 ± 15 | 52 | 133 ± 19 | 0.505 |
| Diastolic BP (mmHg) | 23 | 75 ± 9 | 52 | 78 ± 10 | 0.398 |
| **Medication** | | | | | |
| Statins | 23 | 1 (4%) | 52 | 22 (42%) | **<0.001** |
| ACEi | 23 | 4 (17%) | 52 | 26 (50%) | **0.010** |

*Results are based on the cohort of patients with at least two visits (N=75). Continuous variables are reported as “median (interquartile range)”, or as “mean ± standard deviation”, as appropriate, with p-values from Mann-Whitney U tests. Categorical variables are reported as “N (%)”, with p-values from Fisher’s exact tests, unless stated otherwise. Bold p-values are significant at p<0.05.* ***^a^*** *p-Value from Mann-Whitney U test, as the factor is ordinal. ACEi=Angiotensin-converting enzyme inhibitors, ACR=Albumin:creatinine ratio, BP=Blood pressure, eGFR=Estimated glomerular filtration rate, NT-proBNP=N-terminal-pro-B-type natriuretic peptide.*

***Supplemental Table S5b – TTE parameters at first visit by disease-modifying therapy***

|  | **Disease-Modifying Therapy at First Visit** | | | |  |
| --- | --- | --- | --- | --- | --- |
|  | **No** | | **Yes** | | **p-**  **Value** |
|  | ***N*** | ***Statistic*** | ***N*** | ***Statistic*** |  |
| **Ventricular Dimensions, Volume and Function** | | | | | |
| LVIVSd (cm) | 23 | 1.0 (0.9, 1.1) | 51 | 1.5 (1.2, 1.7) | **<0.001** |
| LVEDd (cm) | 23 | 4.3 ± 0.2 | 51 | 4.5 ± 0.5 | 0.093 |
| LVPWd (cm) | 23 | 1.0 ± 0.1 | 51 | 1.3 ± 0.3 | **<0.001** |
| LVESd (cm) | 23 | 2.8 ± 0.3 | 44 | 2.9 ± 0.5 | 0.560 |
| LVEDvol 2D (ml) | 21 | 86 ± 27 | 33 | 88 ± 27 | 0.811 |
| LVESvol 2D (ml) | 21 | 34 (27, 40) | 33 | 32 (25, 39) | 0.607 |
| LVEF-BP (%) | 19 | 64 ± 4 | 34 | 62 ± 6 | 0.294 |
| MAPSE (cm) | 21 | 15 ± 3 | 44 | 13 ± 4 | **0.035** |
| TAPSE (cm) | 23 | 23 ± 3 | 43 | 21 ± 5 | 0.197 |
| LVM (g) | 23 | 142 (136, 151) | 51 | 248 (187, 319) | **<0.001** |
| LVMi (g/m^2^) | 23 | 80 (68, 91) | 50 | 134 (96, 168) | **<0.001** |
| LV MWT (cm) | 23 | 1.0 ± 0.2 | 51 | 1.5 ± 0.4 | **<0.001** |
| GLS A4C (%) | 13 | -19.2 ± 3.4 | 18 | -16.4 ± 2.7 | **0.005** |
| **Doppler Studies and Ratios** | | | | | |
| TDI Lat s (cm/s) | 23 | 10 ± 2 | 51 | 9 ± 3 | **<0.001** |
| TDI Lat e (cm/s) | 23 | 15 ± 4 | 51 | 9 ± 4 | **<0.001** |
| TDI Lat a (cm/s) | 22 | 9 ± 2 | 47 | 8 ± 2 | 0.219 |
| TDI Sep s (cm/s) | 23 | 9 ± 1 | 50 | 7 ± 2 | **0.001** |
| TDI Sep e (cm/s) | 23 | 12 ± 3 | 49 | 7 ± 3 | **<0.001** |
| TDI Sep a (cm/s) | 23 | 8 ± 1 | 45 | 8 ± 2 | 0.299 |
| TDI RV s (cm/s) | 21 | 14 ± 2 | 36 | 13 ± 3 | 0.191 |
| TDI RV e (cm/s) | 21 | 14 ± 3 | 35 | 10 ± 3 | **<0.001** |
| TDI RV a (cm/s) | 21 | 11 ± 3 | 32 | 13 ± 3 | 0.195 |
| MV E max (m/s) | 23 | 85 ± 19 | 50 | 77 ± 20 | 0.163 |
| MV A max (m/s) | 23 | 55 ± 14 | 47 | 70 ± 19 | **0.003** |
| E/A | 23 | 1.66 ± 0.58 | 47 | 1.18 ± 0.49 | **<0.001** |
| AV Vmax (m/s) | 23 | 131 (116, 150) | 48 | 131 (119, 150) | 0.941 |
| LVOT Vmax (m/s) | 22 | 109 (93, 117) | 47 | 104 (93, 125) | 0.752 |
| TV E (m/s) | 21 | 54 ± 14 | 40 | 55 ± 16 | 0.654 |
| TV A (m/s) | 21 | 38 ± 12 | 35 | 46 ± 11 | **0.010** |
| TR Vmax (cm/s) | 16 | 203 ± 24 | 19 | 224 ± 38 | 0.159 |
| **Atrial Dimensions and Function** | | | | | |
| LAV (ml) | 22 | 39 (24, 47) | 47 | 48 (37, 72) | **0.001** |
| LA Vi (ml/m^2^) | 22 | 20 (16, 23) | 41 | 26 (20, 33) | **0.001** |
| LA GCS (%) | 18 | -26 (-48, -10) | 39 | -36 (-52, -14) | 0.503 |
| LA GLS (%) | 18 | -33 (-36, -16) | 39 | -27 (-41, -15) | 0.718 |
| LA EF (%) | 18 | 50 ± 20 | 39 | 52 ± 20 | 0.643 |
| LA FAC (%) | 18 | 38 ± 17 | 39 | 39 ± 17 | 0.797 |

*Results are based on the cohort of patients with at least two visits (N=75). Data are reported as “median (interquartile range)”, or as “mean ± standard deviation”, as appropriate, with p-values from Mann-Whitney U tests. Bold p-values are significant at p<0.05. Abbreviations for TTE parameters are defined in* ***Supplemental Table S1****.*

***Supplemental Table S6a – Longitudinal trends in TTE parameters and physiological / biochemical markers by disease-modifying therapy***

|  | **Disease-Modifying Therapy at First Visit** | | | |
| --- | --- | --- | --- | --- |
|  | **No** | | **Yes** | |
|  | ***Gradient (95% CI)*** | ***p-Value*** | ***Gradient (95% CI)*** | ***p-Value*** |
| **Ventricular Dimensions, Volume and Function** | | | | |
| LVIVSd (% per Year) **^a^** | -0.3% (-2.2%, 1.5%) | 0.715 | 0.1% (-0.9%, 1.1%) | 0.841 |
| LVEDd (cm per Year) | -0.05 (-0.09, -0.02) | **0.002** | 0.02 (-0.01, 0.05) | 0.213 |
| LVPWd (cm per Year) | 0.01 (0.00, 0.02) | 0.131 | 0.00 (-0.01, 0.02) | 0.514 |
| LVESd (cm per Year) | -0.03 (-0.06, 0.00) | **0.050** | 0.00 (-0.02, 0.03) | 0.777 |
| LVEDvol 2D (% per Year) **^a^** | -1.0% (-4.1%, 2.1%) | 0.520 | 1.2% (-0.3%, 2.7%) | 0.112 |
| LVESvol 2D (% per Year) **^a^** | -2.6% (-4.5%, -0.6%) | **0.009** | 2.1% (0.0%, 4.3%) | **0.048** |
| LVEF-BP (pp per Year) **^b^** | -0.19 (-0.57, 0.20) | 0.341 | -0.15 (-0.46, 0.15) | 0.324 |
| MAPSE (% per Year) **^a^** | -2.3% (-4.5%, -0.1%) | **0.039** | -0.8% (-2.2%, 0.7%) | 0.295 |
| TAPSE (cm per Year) | -0.52 (-0.92, -0.12) | **0.011** | -0.21 (-0.45, 0.04) | 0.096 |
| LVM (% per Year) **^a^** | -1.5% (-3.5%, 0.6%) | 0.170 | 1.0% (-0.4%, 2.4%) | 0.163 |
| LVMi (% per Year) **^a^** | -0.8% (-2.7%, 1.1%) | 0.399 | 1.1% (-0.3%, 2.5%) | 0.111 |
| LV MWT (cm per Year) | 0.00 (-0.01, 0.02) | 0.862 | 0.00 (-0.01, 0.01) | 0.961 |
| GLS A4C (pp per Year) **^b c^** | 0.00 (-0.33, 0.33) | 1.000 | 0.36 (0.04, 0.67) | **0.025** |
| **Doppler Studies and Ratios** | | | | |
| TDI Lat s (cm/s per Year) | -0.22 (-0.35, -0.09) | **0.001** | -0.04 (-0.17, 0.09) | 0.566 |
| TDI Lat e (% per Year) **^a^** | -4.5% (-7.1%, -1.9%) | **<0.001** | 0.4% (-1.4%, 2.4%) | 0.650 |
| TDI Lat a (cm/s per Year) | -0.02 (-0.20, 0.15) | 0.786 | -0.15 (-0.27, -0.02) | **0.022** |
| TDI Sep s (cm/s per Year) | -0.11 (-0.22, 0.00) | 0.050 | -0.12 (-0.20, -0.04) | **0.002** |
| TDI Sep e (% per Year) **^a^** | -4.7% (-6.2%, -3.2%) | **<0.001** | -1.0% (-2.8%, 0.8%) | 0.261 |
| TDI Sep a (cm/s per Year) | 0.00 (-0.21, 0.21) | 0.990 | -0.07 (-0.18, 0.03) | 0.171 |
| TDI RV s (cm/s per Year) | -0.22 (-0.41, -0.03) | **0.021** | -0.11 (-0.28, 0.06) | 0.205 |
| TDI RV e (cm/s per Year) | -0.40 (-0.62, -0.19) | **<0.001** | 0.08 (-0.14, 0.31) | 0.477 |
| TDI RV a (cm/s per Year) | 0.00 (-0.37, 0.37) | 0.996 | -0.17 (-0.44, 0.10) | 0.218 |
| MV E max (m/s per Year) | -1.33 (-2.72, 0.06) | 0.061 | 0.54 (-0.10, 1.19) | 0.096 |
| MV A max (m/s per Year) | 1.07 (0.16, 1.99) | **0.022** | 0.55 (-0.83, 1.93) | 0.434 |
| E/A (% per Year) **^a^** | -4.1% (-6.7%, -1.5%) | **0.002** | 0.6% (-1.6%, 2.9%) | 0.614 |
| AV Vmax (% per Year) **^a^** | -0.9% (-2.3%, 0.6%) | 0.240 | 2.4% (0.9%, 4.0%) | **0.002** |
| LVOT Vmax (% per Year) **^a^** | 0.6% (-0.5%, 1.7%) | 0.288 | -0.4% (-1.8%, 1.1%) | 0.627 |
| TV E (m/s per Year) | -0.50 (-1.98, 0.98) | 0.506 | -0.19 (-0.92, 0.54) | 0.612 |
| TV A (m/s per Year) | 0.63 (-0.72, 1.98) | 0.358 | 0.26 (-0.62, 1.14) | 0.567 |
| TR Vmax (cm/s per Year) | 4.0 (1.4, 6.5) | **0.002** | 2.6 (-0.5, 5.6) | 0.096 |

*Results are from generalised estimating equation models on patients with at least two visits (N=52 on disease-modifying therapy at the first visit; N=23 not on therapy), with the stated parameter as the dependent variable, and the timing of the visit as a covariate. Separate models were produced for subgroups defined by whether patients were on disease-modifying therapy at the first visit. Gradients are reported alongside 95% confidence intervals (95% CIs), and bold p-values are significant at p<0.05. Definitions of the TTE parameter abbreviations are reported in* ***Supplemental Table S1****. Analyses included only those TTEs for which the stated parameter was recorded – see* ***Tables 3 and 4*** *for the included sample sizes.* ***^a^*** *Values were log_2_-transformed prior to analysis, in order to improve model fit; the resulting gradients were then anti-logged, and converted to percentage increases per year.* ***^b^*** *For TTE parameters that are measured as percentages, the gradients represent percentage point (pp) increases per year; for example a gradient of 1 would represent an increase from 4% to 5% in one year.* **^c^** *GCS and GLS values were recorded as negative percentages; hence, the positive gradient indicates a longitudinal trend towards zero.*

***Supplemental Table S6b – Longitudinal trends in TTE parameters and physiological / biochemical markers by disease-modifying therapy***

|  | **Disease-Modifying Therapy at First Visit** | | | |
| --- | --- | --- | --- | --- |
|  | **No** | | **Yes** | |
|  | ***Gradient (95% CI)*** | ***p-Value*** | ***Gradient (95% CI)*** | ***p-Value*** |
| **Atrial Dimensions and Function** | | | | |
| LAV (% per Year) **^a^** | 1.4% (-2.3%, 5.3%) | 0.464 | 2.3% (0.0%, 4.7%) | 0.052 |
| LA Vi (% per Year) **^a^** | 1.0% (-2.2%, 4.3%) | 0.562 | 3.8% (1.5%, 6.2%) | **0.001** |
| LA GCS (% per Year) **^a c d^** | 3.7% (-6.1%, 14.6%) | 0.473 | 3.9% (-0.8%, 8.8%) | 0.106 |
| LA GLS (% per Year) **^a c d^** | 1.5% (-5.0%, 8.5%) | 0.652 | 3.6% (0.4%, 7.0%) | **0.027** |
| LA EF (pp per Year) **^b^** | -0.44 (-2.00, 1.11) | 0.576 | -1.10 (-2.05, -0.15) | **0.023** |
| LA FAC (pp per Year) **^b^** | -0.51 (-1.83, 0.82) | 0.452 | -0.88 (-1.54, -0.22) | **0.009** |
| **Physiological and Biochemical Markers** | | | | |
| Haemoglobin (g/l per Year) | 0.51 (-0.28, 1.30) | 0.204 | -1.00 (-1.93, -0.06) | **0.037** |
| eGFR (ml/min/1.73m^2^ per Year) **^e^** | 0.3 (-0.8, 1.5) | 0.556 | -2.7 (-3.7, -1.7) | **<0.001** |
| Troponin-I (% per Year) **^a^** | -9.6% (-27.4%, 12.6%) | 0.368 | 7.4% (-2.2%, 17.9%) | 0.136 |
| Troponin-T (% per Year) **^a^** | 11.3% (-3.8%, 28.8%) | 0.150 | 11.5% (1.9%, 22.1%) | **0.018** |
| NT-proBNP (% per Year) **^a^** | 0.0% (-8.3%, 9.1%) | 0.993 | 12.2% (4.8%, 20.3%) | **0.001** |
| ACR (% per Year) **^a^** | 4.8% (-1.5%, 11.5%) | 0.135 | 3.7% (-3.0%, 10.9%) | 0.287 |
| Urine Protein (% per Year) **^a^** | 2.4% (-11.5%, 18.6%) | 0.748 | 4.5% (-2.2%, 11.6%) | 0.197 |
| Cholesterol (mmol/l per Year) | 0.02 (-0.07, 0.11) | 0.680 | -0.07 (-0.12, -0.02) | **0.003** |
| Systolic BP (mmHg per Year) | 0.76 (-0.37, 1.88) | 0.187 | -0.75 (-1.35, -0.15) | **0.015** |
| Diastolic BP (mmHg per Year) | 0.58 (-0.05, 1.22) | 0.071 | 0.04 (-0.43, 0.50) | 0.879 |

*Results are from generalised estimating equation models on patients with at least two visits (N=52 on disease-modifying therapy at the first visit; N=23 not on therapy), with the stated parameter as the dependent variable, and the timing of the visit as a covariate. Separate models were produced for subgroups defined by whether patients were on disease-modifying therapy at the first visit. Gradients are reported alongside 95% confidence intervals (95% CIs), and bold p-values are significant at p<0.05. Definitions of the TTE parameter abbreviations are reported in* ***Supplemental Table S1****. Analyses included only those TTEs for which the stated parameter was recorded – see* ***Tables 3 and 4*** *for the included sample sizes.* ***^a^*** *Values were log_2_-transformed prior to analysis, in order to improve model fit; the resulting gradients were then anti-logged, and converted to percentage increases per year.* ***^b^*** *For TTE parameters that are measured as percentages, the gradients represent percentage point (pp) increases per year; for example a gradient of 1 would represent an increase from 4% to 5% in one year.* **^c^** *GCS and GLS values were recorded as negative percentages; hence, the positive gradient indicates a longitudinal trend towards zero. ^d^ To normalise the negatively skewed distribution, absolute values were taken, to convert values from negative to positive, which were subsequently log_2_-transformed; the direction of the resulting gradient was then reversed to reflect the original scale. ^e^* *Analysis of eGFR excluded patients with CKD stage 4 or 5 at the first visit (N=5), in order to improve model fit. ACR=Albumin:creatinine ratio, BP=Blood pressure, eGFR=Estimated glomerular filtration rate, NT-proBNP=N-terminal-pro-B-type natriuretic peptide.*

***Supplemental Figure S1 – Correlations between trends in NT-proBNP and trends in TTE parameters***

*Points represent the gradients for individual patients, calculated as described in* ***Supplemental Table S3****. Trend lines are from regression models, with the gradient in the stated TTE parameter as the dependent variable, and the NT-proBNP gradient as a covariate. For parameters that were log_2_-transformed prior to analysis (see* ***Table 3*** *for details), the gradients for individual patients and trend line from the resulting regression model were converted to percentage increases per year for the plots, to simplify interpretation. Definitions of the TTE parameter abbreviations are reported in* ***Supplemental Table S1****. pp=percentage point, rho=Spearman’s rank correlation coefficient*


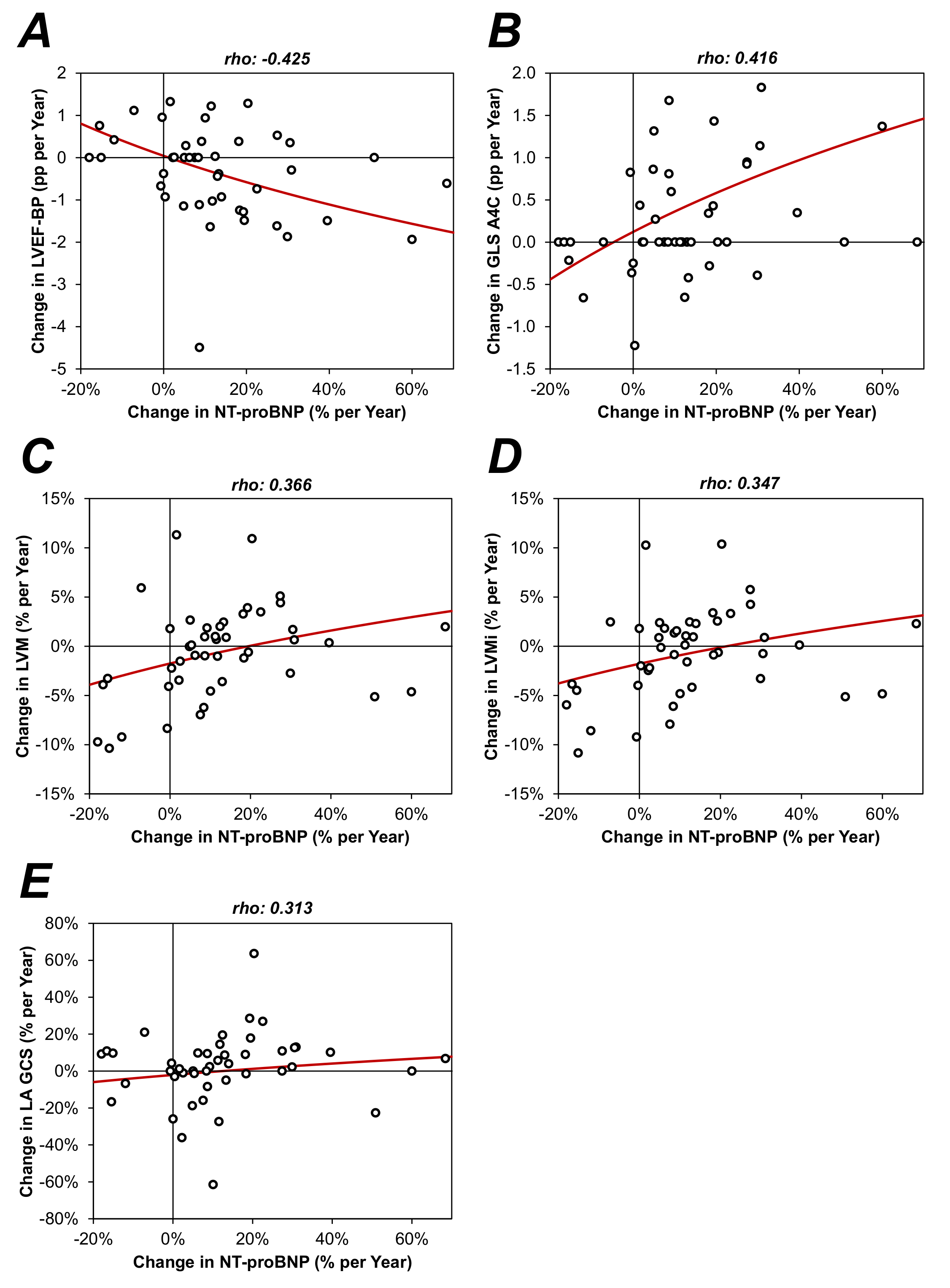

Supplement: Supplemental Table [file mmc1.docx]
